# Quantitation of small intestinal permeability during normal human drug absorption

**DOI:** 10.1186/2050-6511-14-34

**Published:** 2013-06-24

**Authors:** David G Levitt

**Affiliations:** 1 Department of Integrative Biology and Physiology, University of Minnesota, 6-125 Jackson Hall, 321 Church St. S. E, Minneapolis, MN 55455, USA

## Abstract

**Background:**

Understanding the quantitative relationship between a drug’s physical chemical properties and its rate of intestinal absorption (QSAR) is critical for selecting candidate drugs. Because of limited experimental human small intestinal permeability data, approximate surrogates such as the fraction absorbed or Caco-2 permeability are used, both of which have limitations.

**Methods:**

Given the blood concentration following an oral and intravenous dose, the time course of intestinal absorption in humans was determined by deconvolution and related to the intestinal permeability by the use of a new 3 parameter model function (“Averaged Model” (AM)). The theoretical validity of this AM model was evaluated by comparing it to the standard diffusion-convection model (DC). This analysis was applied to 90 drugs using previously published data. Only drugs that were administered in oral solution form to fasting subjects were considered so that the rate of gastric emptying was approximately known. All the calculations are carried out using the freely available routine PKQuest Java (http://www.pkquest.com) which has an easy to use, simple interface.

**Results:**

Theoretically, the AM permeability provides an accurate estimate of the intestinal DC permeability for solutes whose absorption ranges from 1% to 99%. The experimental human AM permeabilities determined by deconvolution are similar to those determined by direct human jejunal perfusion. The small intestinal pH varies with position and the results are interpreted in terms of the pH dependent octanol partition. The permeability versus partition relations are presented separately for the uncharged, basic, acidic and charged solutes. The small uncharged solutes caffeine, acetaminophen and antipyrine have very high permeabilities (about 20 x 10^-4^ cm/sec) corresponding to an unstirred layer of only 45 μm. The weak acid aspirin also has a large AM permeability despite its low octanol partition at pH 7.4, suggesting that it is nearly completely absorbed in the first part of the intestine where the pH is about 5.4.

**Conclusions:**

The AM deconvolution method provides an accurate estimate of the human intestinal permeability. The results for these 90 drugs should provide a useful benchmark for evaluating QSAR models.

## Background

Despite the multitude of publications describing the different factors that affect the rate of intestinal absorption of drugs, there is only limited experimental data for the human small intestinal permeability of the thousands of drugs that are orally absorbed. The quantitative structure activity relationship (QSAR) between a drug’s physical chemical properties and its rate of intestinal absorption is obviously of great importance in selecting candidate drugs. The standard approach is to relate some property of the drug (e.g. octanol/water partition, Caco-2 cell permeability, etc.) to the fraction absorbed in humans [1,2]. Although the fraction absorbed is a useful clinical parameter [3], it is a crude measure of permeability. Since most successful drugs are nearly 100% absorbed, they cannot provide any quantitative data about their relative permeability. Furthermore, the fraction absorbed may be influenced in uncertain ways by factors such as intestinal metabolism or large intestinal absorption.

More recently, there have been direct measurements of human small intestinal permeability using the regional perfusion technique. In a recent communication, Dahan, Lennernas and Amidon [4] discuss the various reasons why these measurement of “…jejunal permeability (alone) may not always adequately predict” the fraction absorbed. This includes small intestinal heterogeneity (such as variations in pH and membrane transport systems) and large intestinal absorption. In addition, the regional perfusion conditions used in these measurements may differ from the normal physiological conditions. For example, the high pressure and volume in the perfusion system may increase access to the intervillous space allowing increased paracellular transport of PEG markers [5].

This paper describes a new approach to measuring human intestinal permeability during normal drug absorption. It is well recognized that the time course of intestinal absorption can be determined from deconvolution of the plasma concentrations following oral and intravenous input in the same subject. There are a variety of mathematical approaches to this deconvolution [6]. Some care is required in this procedure because random errors in the plasma concentration data can lead to non-physiological fluctuations or negative values in the predicted absorption rate. The simplest procedures assume that the absorption can be described by some simple function (e.g. 3 parameter gamma [6] or Hill function [7]) which is then adjusted to give the best fit to the oral plasma absorption curve. More sophisticated approaches use generalized functions with varying numbers of parameters [8,9]. This absorption function must then be interpreted in terms of the intestinal permeability. This is difficult because intestinal transit, dispersion and absorption is complicated and poorly understood. The most widely used quantitative model of intestinal absorption is the “compartmental absorption and transit” (CAT) model which has been incorporated into the commercial program GastroPlus™ [10,11]. This CAT model describes the small intestine in terms of 7 sequential well mixed compartments with passive absorption (determined by the permeability) and one way transport in the aboral direction. Because the solution of this model’s equations requires numerical calculations and does not have an analytical solution, it cannot be easily adapted for the deconvolution approach.

In this paper a new 3 parameter function (“Averaged Model” (AM)) that accurately mimics the transit, dispersion and absorption of the small intestine is used to determine the intestinal permeability by deconvolution. The range of validity of this AM model is evaluated by comparing it with the more exact diffusion convection model (DC). This AM procedure is then applied to published data to determine the human intestinal permeability of 90 drugs. The main criterion for the selection of drugs for this analysis is that they were administered as an oral solution in order to eliminate the ambiguity and variability in the rate of gastric emptying.

## Methods

### Numerical solution of the Diffusion-Convection (DC) model equations

Ni et. al. [12] have described a model of intestinal transit which combines convection, dispersion and absorption (DC model). The main assumption is that there is an equal volume flow into and out of each intestinal region so that the cross-sectional area (radius = r) and the convective flow (F) remains constant as the solute spreads along the intestine by convection and dispersion. The differential equation describing this DC model is:

(1) $$\pi r^{2}\frac{\partial c}{\partial t}=\pi r^{2}D\frac{\partial^{2}c}{\partial x^{2}}-F\frac{\partial c}{\partial x}-2\pi \text{rPc}$$

The left hand side is the time dependent change in the concentration c(x,t) (where x is the distance from the pyloric sphincter). The first term on the right is the dispersive mixing, the second is the convective flow and the third is the absorption term where r is the intestinal radius (cm), D is the dispersion coefficient (cm^2^/sec), F is the volume flow (cm^3^/sec) and P is the permeability (cm/sec).

Ni et. al. [12] derived an exact analytical solution to Equation (1) that assumes as a boundary condition an exponential concentration at x = 0. This condition is not physiological because it implies that, in addition to the convective flux out of the stomach, there is also a non-physiological dispersive flux both out of and into the stomach (and out of and into the large intestine). For this reason the analytical solution will not be used here and, instead, a finite difference numerical approximation to Equation (1) will be used in which there is only a convective flux from the stomach to the small intestine and from the small intestine to the large intestine. (Also, the numerical solution is computationally much faster than the analytical solution). The small intestine is divided into N equal sections with the following difference equations:

(2) $$\begin{array}{l} i=1:\;\Delta V\;\frac{\mathrm{dc}\left[1\right]}{\mathrm{dt}}=I_{G}\left(t\right)-(F+\Delta P+\mathrm{De})\;c[1]+\mathrm{De}\;c[2]\\ 0<i<N:\;\Delta V\;\frac{\mathrm{dc}\left[i\right]}{\mathrm{dt}}=\left(F+\mathrm{De}\right)\;c[i-1]-(F+\Delta P+2\;\mathrm{De})\;c[i]+\mathrm{De}\;c[i+1]\\ i=N:\;\Delta V\;\frac{\mathrm{dc}\left[N\right]}{\mathrm{dt}}\;=\left(F+\mathrm{De}\right)\;c[N-1]-(F+\mathrm{De}+\Delta P)\;c[N]\; \end{array}$$

where c[i] is the concentration in the ith compartment at time t, I_G_(t) is the rate of gastric emptying into the intestine, r = intestinal radius, L = intestinal length, S = surface area = 2πrL, V = volume = πr^2^L, ∆P = PS/N, ∆V = V/N and De = πr^2^DN/L_._ The rate E_DC_(t) that the unabsorbed solute exits the small intestine and passes into the large intestine is:

(3) $$E_{\mathrm{DC}}\left(t\right)=F\;c\left[N\right]$$

The cumulative amount A_DC_(t_i_) that has entered the large intestine at time t_i_ = i ∆t is:

(4) $$A_{\mathrm{DC}}\left(t_{i}\right)=\sum_{j=1}^{i}\;E_{\mathrm{DC}}\left(t_{j}\right)\;\Delta t$$

The absorption rate R_DC_(t) at time t_i_ is:

(5) $$R_{\mathrm{DC}}\left(t_{i}\right)=\Delta P\;\sum_{i=1}^{N}c\left[i\right]$$

Gastric emptying in humans of non-caloric fluids is approximately exponential with a half time of about 15 minutes [13,14] and it will be assumed that I_G_(t) is exponential:

(6) $$I_{G}\left(t\right)=FC_{0}\text{exp}\left(-t/T_{G}\right)$$

where T_G_ is the time constant for gastric emptying, C_0_ is the gastric concentration at t = 0 and FC_0_ = Dose/T_G_. In addition, the parameters D, F and P will be described in terms of 3 other time constants:

(7) $$T_{P}=r/\left(2P\right)\;T_{F}=V/F\;T_{D}=L^{2}/\left(2D\right)$$

Equation (2) is solved numerically using N = 50 and the Rosenbrock method as implemented in Maple (Maplesoft™). Some of the figures shown here are Maple plots.

### Derivation and description of the “Averaged Model (AM)”

The DC equation (Equation (1)) has the interesting property that, if the drug is completely absorbed in the small intestine and the amount entering the large intestine can be neglected, it has the same kinetics as a well stirred compartment. This can be seen by integrating Equation (1) over x from 0 (pyloric sphincter) to x = L (the ileocecal junction):

(8) $$\begin{array}{l} \;\pi r^{2}L\frac{\mathrm{dC}}{\mathrm{dt}}=\;I_{0}\left(t\right)\;-I_{L}(t)\;-2\text{πrLPC}\\ C\;=\;\left(1/L\right)\int_{0}^{L}c\left(x,t\right)\mathrm{dx}\;I_{0}(t)\;=\;-\pi r^{2}D\frac{\mathrm{dc}\left(0,t\right)}{\mathrm{dx}}\;+\;\mathrm{Fc}(0,t)\;\\ I_{L}(t)\;=\;-\pi r^{2}D\frac{\mathrm{dc}\left(L,t\right)}{\mathrm{dx}}\;+\mathrm{Fc}(L,t)\; \end{array}$$

where C is the average intestinal concentration and I_0_(t) and I_L_(t) are the inflow and outflow rates. If the outflow term I_L_(t) is negligible, then this equation reduces to:

(9) $$\;V\frac{\mathrm{dC}}{\mathrm{dt}}=I_{0}\left(t\right)-\mathrm{PS}\;C$$

This is identical to the case of a well mixed compartment of volume V with arbitrary input I_0_(t). Assuming that I_0_(t) = I_G_(t) (Equation (6)) and solving the differential Equation (9) one obtains the “averaged model” (AM) equation for the case of 100% absorption:

(10) $$C\left(t\right)=\left(\text{Dose}/V\right)\;T_{P}\;\left[\text{exp}\left(-t/T_{G}\right)\;-\text{exp}\left(-t/T_{P}\right)\right]/\left(T_{G}-T_{P}\right)$$

where T_P_ and T_G_ are the permeability and gastric emptying time constants (Equation (6) and (7)). The rate of absorption (R(t)) from the small intestine is:

(11) $$R\left(t\right)=P\;S\;C\left(t\right)=\mathrm{Dose}\;\left[\text{exp}\left(-t/T_{G}\right)\;-\text{exp}\left(-t/T_{P}\right)\right]/\left(T_{G}-T_{P}\right)$$

This AM R(t) is identical to the absorption rate for the DC model for the case where all the solute is absorbed (I_L_(t) = 0, Equation (8)). It should be emphasized that although Equation (9) is similar to the well-mixed equation it is not physically equivalent because C is the average concentration and it is not assumed that the intestine is well mixed. For example, it would be erroneous to assume that the rate of solute flow into the large intestine was equal to F*C.

As discussed above, Equation (11) is a rigorously accurate description of the intestinal absorption for the DC model only for the case where all of the solute is absorbed in the small intestine. This result can be generalized to the arbitrary permeability case where only a fraction F_A_ of the total Dose is absorbed in the small intestine:

(12) $$\begin{array}{l} R_{M}\left(t\right)=M\;[\text{exp}(-t/T_{G})\;-\text{exp}(-t/T_{P})]/(T_{G}-T_{P})\\ M=F_{A}\;\text{Dose} \end{array}$$

where M is the total amount absorbed. In addition, the relationship between T_P_ and P must be modified for this general case. The C in Equation (8) is based on the assumption of 100% absorption. If, for example, only 50% were absorbed the actual concentration would be twice this value of C and the value of P would be reduced by half. Thus, the general relationship between T_P_ and the averaged model intestinal permeability (P_M_) is:

(13) $$P_{M}=F_{A}\;P=F_{A}\;r/\left(2T_{P}\right)$$

The amount absorbed (A_M_(t)) as a function of time is:

(14) $$\begin{array}{ll} A_{M}\left(t\right) & =\int_{0}^{t}R_{M}\left(\tau \right)\mathrm{dt}=M\{1+[\;T_{P}\text{exp}\left(-t/T_{P}\right)\\ & -T_{G}\text{exp}\left(-t/T_{G}\right)\;]/\left(T_{G}-T_{P}\right)\;\} \end{array}$$

These AM model expressions for the intestinal absorption rate R_M_(t) and P_M_ are only approximations to the exact DC model for this general case where not all the solute is absorbed. The range of validity of this approximation will be evaluated by comparing it to the DC model for a range of experimental parameters (see Results, Comparison of DC and AM models).

R_M_ and M represent the rate and total amount absorbed across the small intestinal epithelial luminal membrane. Assuming a linear system, the rate of solute entering the systemic circulation (R_SM_) is:

(15) $$\begin{array}{l} R_{\mathrm{SM}}\left(t\right)=M_{S}\;[\text{exp}(-t/T_{G})\;-\text{exp}(-t/T_{P})]/(T_{G}-T_{P})\\ M_{S}=F_{A}\left(1-E_{H}\right)(1-E_{I})\;\mathrm{Dose} \end{array}$$

where E_H_ and E_I_ are the hepatic and intestinal extraction ratios [15]. The hepatic extraction (E_H_) can be estimated from the liver blood flow (Q_H_) [15] and the whole blood liver clearance (Cl_H_):

(16) $$E_{H}=Cl_{H}/Q_{H}$$

The liver clearance (Cl_H_) was estimated by correcting the whole blood clearance following the IV infusion for the fractional renal clearance using data obtained in the same subjects that were used for the permeability estimates.

Equation (15) is a simple 3 parameter function whose parameters (M_S_, T_G_ and T_P_) can be determined experimentally by deconvolution (see below for details) of the blood concentration time course following IV and oral doses. The fraction absorbed (F_A_) can be determined from M_S_ and estimates of E_H_ and E_I_ (Equation (15)). Finally, the AM model intestinal permeability (P_M_) can be determined from F_A_ and T_P_ (Equation (13)).

Equation (15) is symmetrical in T_G_ and T_P_ so that there is an ambiguity in distinguishing the gastric emptying time constant T_G_ from the permeability time constant T_P_. Most of the applications described here will be based on data obtained using oral solutions (not tablets) given to fasting subjects and the time constant that is closest to 10 to 15 minutes will be assumed to be T_G_ .

The theoretical accuracy of the AM model absorption rate RM (Equation 12) was evaluated by comparing it with the exact DC model R_DC_ (Equation 5). A set of the 7 DC parameters (Dose, T_G_, T_P,_ T_F_, T_D_, r, L) were selected and the DC model intestinal absorption rate and fraction absorbed was determined. Then, the AM model parameters (M, T_G_, T_P_, Equation (12)) that provided the best fit to the DC absorption rate were determined by minimizing the following error function using the optimization routine in Maple (Maplesoft™):

(17) $$\mathrm{Error}=\left(1/N\right)\;\sum_{i=1}^{N}{\left(R_{\mathrm{DC}},\left[i\right],-,R_{M},\left(t_{i}\right)\right)}^{2}$$

where t_i_ = i ∆t and comparing the AM model parameters (F_A_, T_P_ and T_G_) with the actual input DC parameters.

### Experimental determination of the averaged model (AM) parameters by deconvolution

The determination of the 3 AM model parameters (M_S_, T_G_ and T_P_) is based on standard procedures that have been described previously [6]. First, the 2 or 3 exponential systemic bolus response function r(t) is determined from the experimental blood concentration time course following the known IV infusion. The blood concentration C_oral_(t) following the oral dose is equal to the convolution of r(t) and the AM model systemic absorption rate R_SM_(t) (Equation (15)):

(18) $$C_{\mathrm{oral}}\left(t\right)=\int_{0}^{t}r\left(t-\tau \right)\;R_{\mathrm{SM}}\left(\tau \right)\;\mathrm{d\tau}$$

The 3 AM model parameters (M_S_, T_G_ and T_P_) are then estimated by finding the parameter set that minimizes the error function:

(19) $$\text{Err}=\sum_{k}\frac{\left|\;C_{\text{oral}}\left(t_{k}\right)-C_{k}\right|}{C_{k}+\text{noise}}$$

where C_k_ is the experimental blood concentration at time t_k_ following the oral dose. The “noise” determines the relative weighting of each data point and can be arbitrarily adjusted but is usually set to 10% of the average blood value. The optimized set of parameters is determined by a non-linear Powell minimization routine [16]. Most of the drugs were administered as oral solutions in fasting subjects and T_G_ was forced to be in the range of 10 to 20 minutes (the normal range for non-caloric fluids [13,14]) and only the two parameters T_P_ and M_S_ were freely adjusted. For a few solutes that were administered as capsules or tablets, all 3 parameters were adjusted.

**Figure 1:**
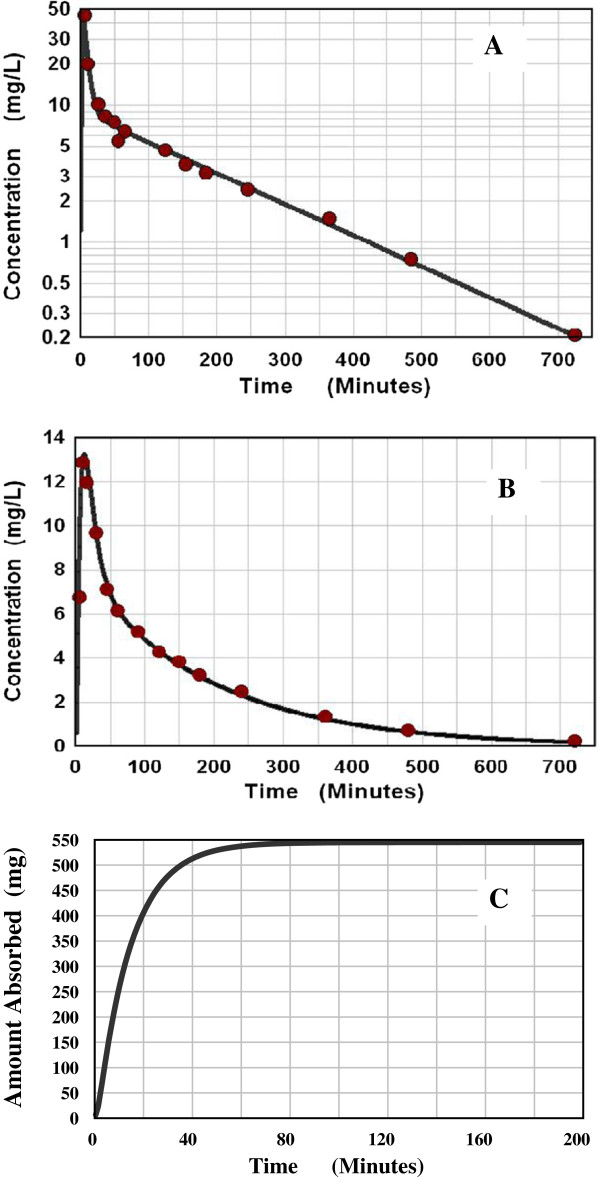
**AM model deconvolution solution for intestinal absorption of acetaminophen.** Figure 1**A** shows a comparison of the experimental blood concentration data points (red circles) following an IV input versus the theoretical blood concentration determined from the 2 exponential systemic response function (line). Figure 1**B** shows a comparison of the experimental blood concentration data points (red circles) following an oral input with the theoretical blood concentration prediction determined from the deconvolution solution of the AM absorption rate function. Figure 1**C** shows the cumulative AM absorption amount as a function of time.

**Figure 2:**
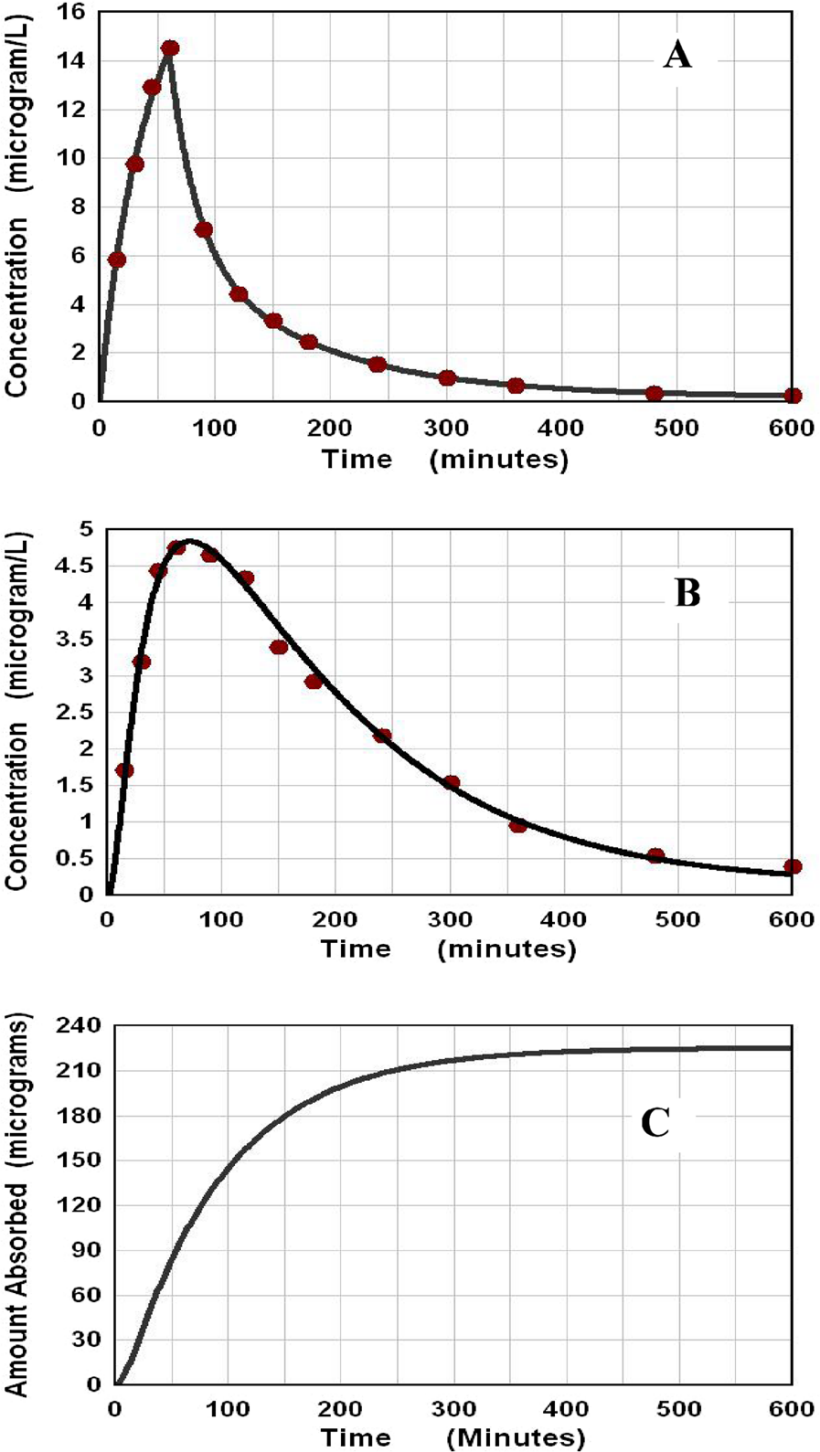
**AM model deconvolution solution for intestinal absorption of risedronate.** See Figure 1 for details.

**Figure 3:**
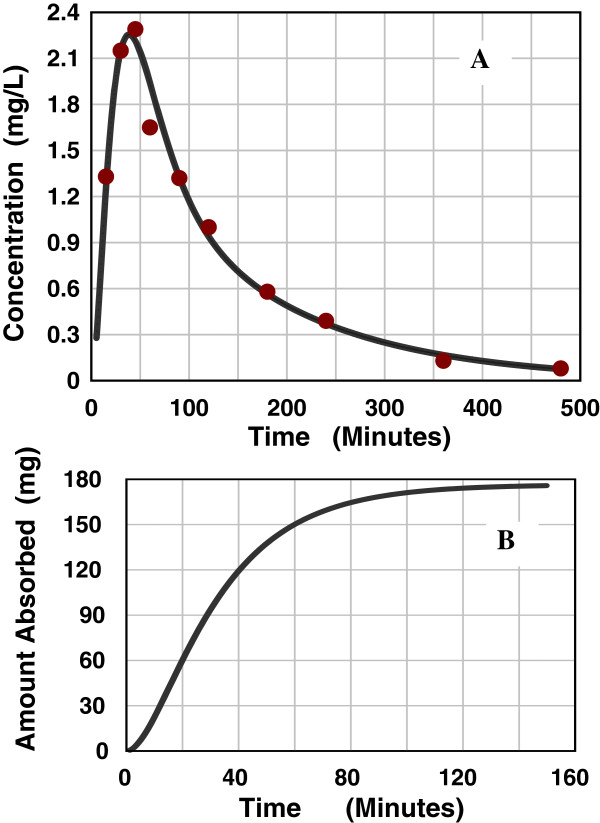
**AM model deconvolution solution for intestinal absorption of cimetidine.** Figure 3**A** shows a comparison of the experimental blood concentration data points (red circles) following an oral input versus the theoretical blood concentration prediction determined from the deconvolution solution of the AM absorption rate function. Figure 3**B** shows the cumulative AM absorption amount as a function of time.

**Figure 4:**
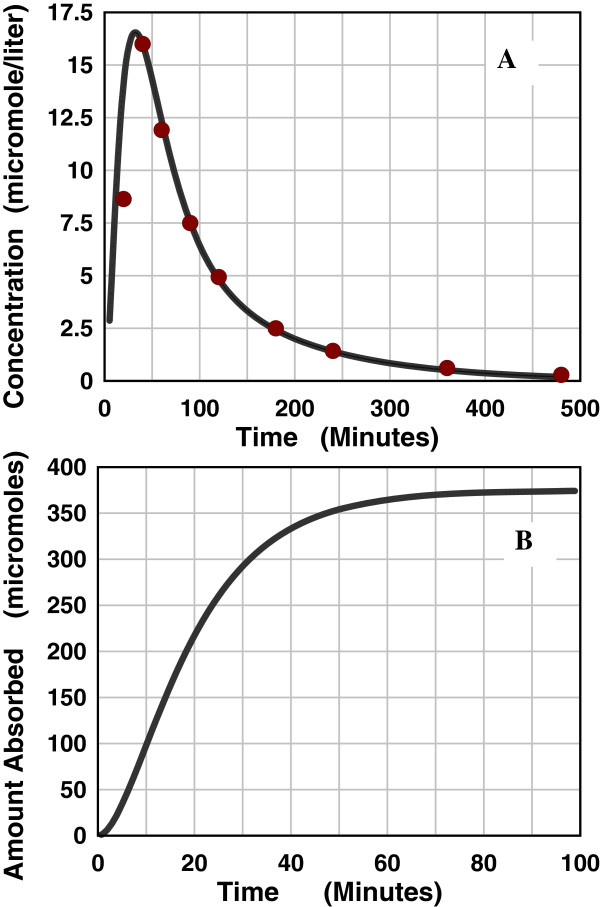
**AM model deconvolution solution for intestinal absorption of acetylcysteine.** Figure 4**A** shows a comparison of the experimental blood concentration data points (red circles) following an oral input versus the theoretical blood concentration prediction determined from the deconvolution solution of the AM absorption rate function. Figure 4**B** shows the cumulative AM absorption amount as a function of time.

These procedures have been implemented in PKQuest Java, a freely distributed software program that has been used previously for pharmacokinetic analysis of more than 30 different solutes in a series of publications [7]. The implementation is designed to be user friendly and simple to use. The user only needs to enter 1) the dose and duration of the constant IV infusion; 2) the experimental blood concentration for the IV dose (which can be copied and pasted from a standard Excel file); and 3) the experimental blood concentration following the oral dose. The program then finds the optimum set of AM parameters. It also outputs 4 plots that are useful for evaluating the results: 1) A comparison of the experimental blood concentration for the IV dose versus the blood concentration predicted by bolus response function (there is usually nearly perfect agreement). 2) The AM model absorption rate as a function of time; 3) AM total absorption as a function of time; and 4) a comparison of the experimental blood concentration following the oral dose versus the AM model prediction (Equation (18)). This last plot is especially useful because it provides the best measure of the quality of the AM model. See Figures 1, 2, 3 and 4 for examples of these plots. PKQuest Java and a detailed tutorial can be freely downloaded from http://www.pkquest.com. Also available for download are the complete data sets for the 90 solutes discussed in this paper. This allows the user to reproduce all of the results.

### Experimental intestinal absorption data

In order to be a candidate for determination of intestinal permeability it was required that the solute met the following 4 conditions: 1) intravenous and oral dose pharmacokinetics in the same subject; 2) the oral dose was in the form of a solution (not tablet) to fasting subjects; 3) the drug’s pharmacokinetics are linear, at least in the concentration range that is investigated; 4) the drug is soluble at the concentrations used in the absorption study. These conditions severely limit the number of experimental results that can be used. Condition #1 is satisfied in only a small fraction of permeability studies. Condition #2 also severely restricts the number of possible candidates because tablets or capsules are used in most oral drug studies. A thorough search of the published literature returned 90 drugs that met these conditions. A few drugs that were administered as tablets have been included if the drug had a high water solubility so that the tablet would be rapidly dissolved and a low permeability (long T_P_) that could not be confused with the T_G_. The results and analyses are summarized in the Excel file that is included in the Additional file 1: “Table 2”. Additional file 1: Table 2 lists the solute, a link to the reference publication, the AM model parameters, a subjective measure of the quality of the AM fit to the data and the calculated permeability. The table includes the ionization behavior of the solute (weak acid, base, neutral or always ionized) in the pH range of 4 to 8 and the pKa if it is a weak base or acid. Also listed is an estimate of the experimental log(octanol/water) partition coefficient at pH 7.4 (log D). For most solutes there are multiple reported values of log D that can vary by as much a log unit. For those solutes which are available on the LOGKOW site maintained by James Sangster, the value listed is an approximate average of the listed values. When necessary, the log Pow values were converted from pH1 to a different pH2 using the following relations (this assumes that only the neutral solute has a finite octanol partition) [17]:

(20) $$\begin{array}{ll} \text{Mono}\;\text{protic}\;\text{base}:\;\text{log}\mathrm{Po}w_{2} & =\text{log}\mathrm{Po}w_{1}+\text{log}\left(1+10^{\left(\text{pKa}-\mathrm{pH}1\right)}\right)\\ & -\text{log}(1+10^{\left(\text{pka}-\mathrm{pH}2\right)})\\ \text{Mono}\;\text{protic}\;\text{acid}:\;\text{log}\mathrm{Po}w_{2} & =\text{log}\mathrm{Po}w_{1}+\text{log}\left(1+10^{\left(\mathrm{pH}1-\text{pKa}\right)}\right)\\ & -\text{log}(1+10^{\left(\mathrm{pH}2-\mathrm{pKa}\right)}) \end{array}$$

The experimental perfused human jejunum permeability [18] and the Caco-2 permeability are also listed in Additional file 1: Table 2 if they were available. The form of the oral dose (solution, tablet, capsule) is listed and solutes which may have solubility limitations are marked in the table. If there is suggestive evidence that the intestinal absorption is protein mediated (either influx or efflux), this is also indicated. The experimental data points were read from the published figures using UN-SCAN-IT (Silk Scientific Corporation).

## Results

### Solution and parameter study of the Diffusion-Convection (DC) model

**Figure 5:**
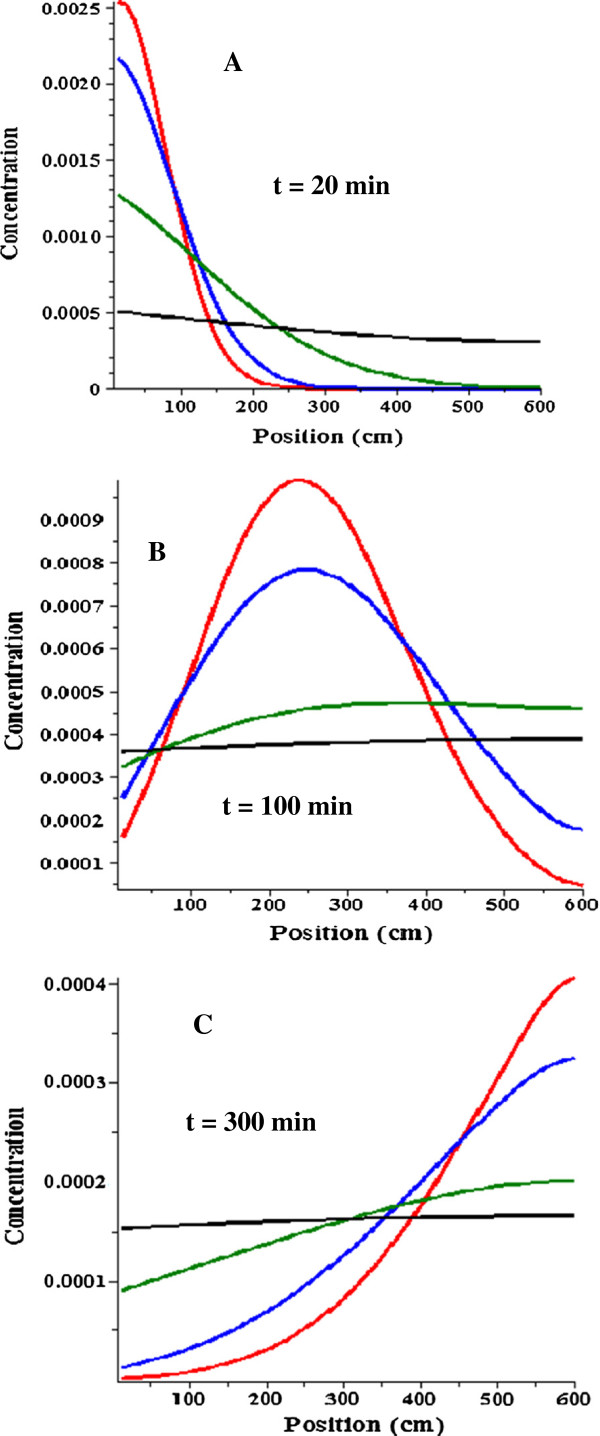
**Diffusion convection concentration profile.** The diffusion convection model concentration as a function of distance from the pyloric sphincter is shown at 20 (Figure 5**A**), 100 (**B**) and 300 minutes (**C**) after administering the oral dose as a bolus to the stomach for an impermeable (P = 0) solute. The profile is shown for 4 different values of the dispersion time constant (T_D_): 2000 (red); 1000 (blue); 200 (green); and 20 minutes (black). For all profiles T_F_ = 200 minutes; T_G_ = 15 minutes; r = 1 cm; L = 600 cm and Dose = 1.

The DC model differential equation (Equation (2)) was solved numerically. Figure 5A, B and C show the DC concentration profile for a non-permeable (∆P = 0) solute at time = 20, 100 and 300 minutes after the oral dose with T_D_ (dispersive transit time) values of 2000 (red curve), 1000 (blue), 200 (green) and 20 (black) minutes. Unless otherwise stated, all of the plots described here have T_G_ = 15 minutes (gastric emptying time constant), T_F_ = 240 minutes (convective small intestinal transit time), N = 50 (there is no significant change in the results for greater N), ∆t = 1 minute, Dose = 1.0, r = 1 cm and L = 600 cm. Since the concentration profile has a strong dependence on T_D_, these plots could be used to estimate the value of T_D_ (and T_F_) in the human if experimental measurements of the concentration profile along the small intestine for impermeable solutes were available. Unfortunately, no such measurements have been reported for humans or other large mammals (they have been made in rats [19]).

**Figure 6:**
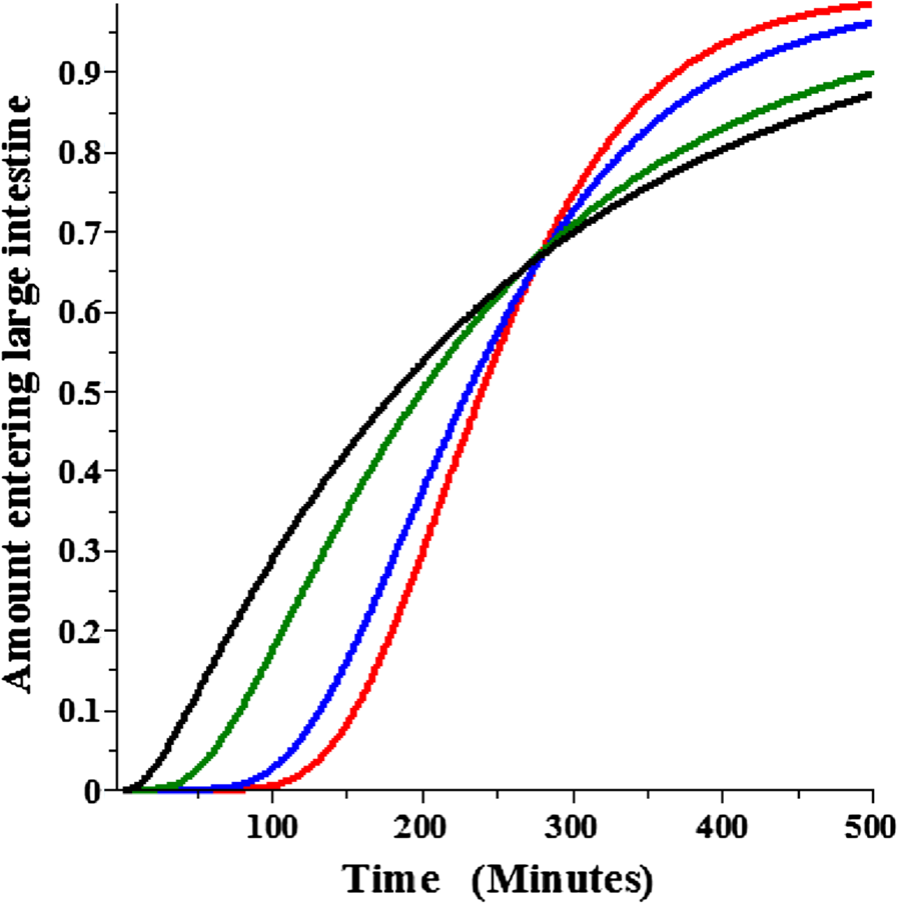
**Diffusion convection small intestinal transit time.** The diffusion convection amount leaving the small intestine and entering the large intestine as a function of time for an impermeable solute. Same conditions as for Figure 5.

The experimental measurement in humans that can be used to estimate T_D_ is the distribution of small intestinal transit times determined from the appearance of some non-permeable label in the large intestine [20]. Figure 6 shows the DC cumulative amount entering the large intestine as a function of time (Equation (4)) for T_D_ = 2000 (red), 1000 (blue), 200 (green) and 20 (black) minutes for the same conditions as in Figure 5. The shape of the curves can be roughly characterized by the time of first appearance of solute and the half time. Caride et. al. [21] reported the time of arrival (time at which a “sustained” increase in breath hydrogen or ^99m^technetium-diethylenetriaminepentaacetic acid was first detected) of about 73 minutes. Based on an extensive literature review, Davis et. al. [22] found an average small intestinal half time of about 240 minutes. From Figure 6, a T_D_ of about 200 minutes provides the best fit to these experimental measurements in humans.

**Figure 7:**
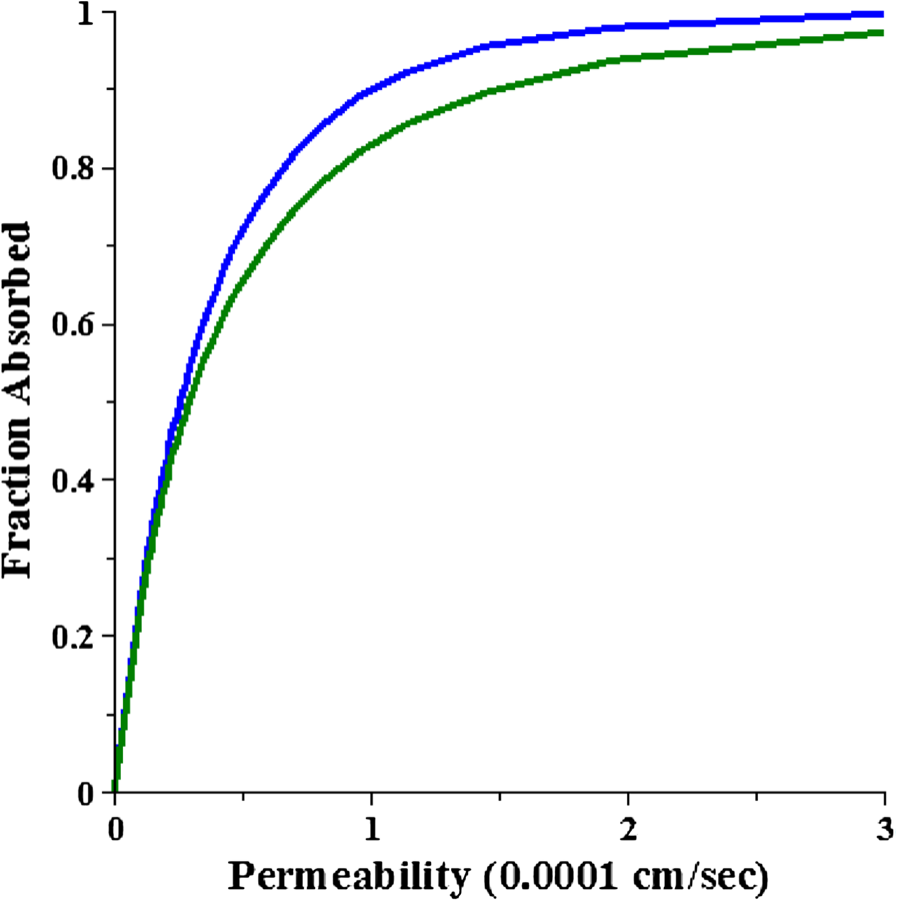
**Permeability dependence of the diffusion convection intestinal absorption.** The fractional absorption of the diffusion convection model as a function of the permeability in units of 10^-4^ cm/sec for a dispersion time constant (T_D_) of 1000 (blue) or 200 minutes (green). The rest of the conditions are the same as in Figure 5.

In the next section, the DC model will be used to evaluate the accuracy of the AM model approximation. The plots in Figures 5 and 6 are for non-permeable solutes. Figure 7 shows a plot of the DC fraction absorbed versus the permeability (10^-4^ cm/sec) for T_D_ = 1000 (blue) and 200 (green) minutes. It can be seen that for the high permeability solutes the fraction absorbed increases by about 5% as T_D_ increases 5 fold (i.e. as the dispersion rate decreases). This produces a small dependence of the error in the AM absorption rate on T_D_ (see below) which is quantitated in the next section.

### Comparison of DC and AM models – theoretical evaluation of accuracy of AM model approximation

**Figure 8:**
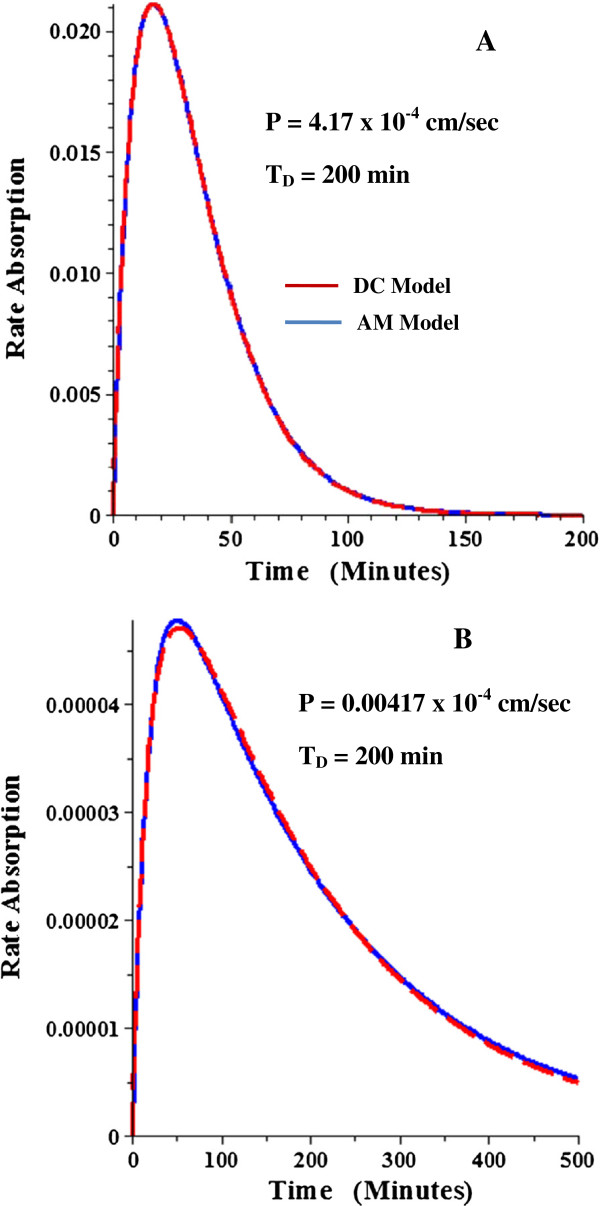
**Comparison of intestinal absorption rate for DC and AM models with T**_**D**_ = **200 minutes.** The DC model rate of absorption (red) was generated for a high permeability solute (P = 4.17 x 10^-4^ cm/sec, Figure 8**A**) and a low permeability solute (P = 0.00417 x 10^-4^ cm/sec, Figure 8**B**) with the rest of conditions the same as in Figure 5. Then the AM model parameters that provided the best fit to the DC model were determined and the AM absorption rate (blue) plotted. See Table 1 for tabulation of the AM parameters.

**Figure 9:**
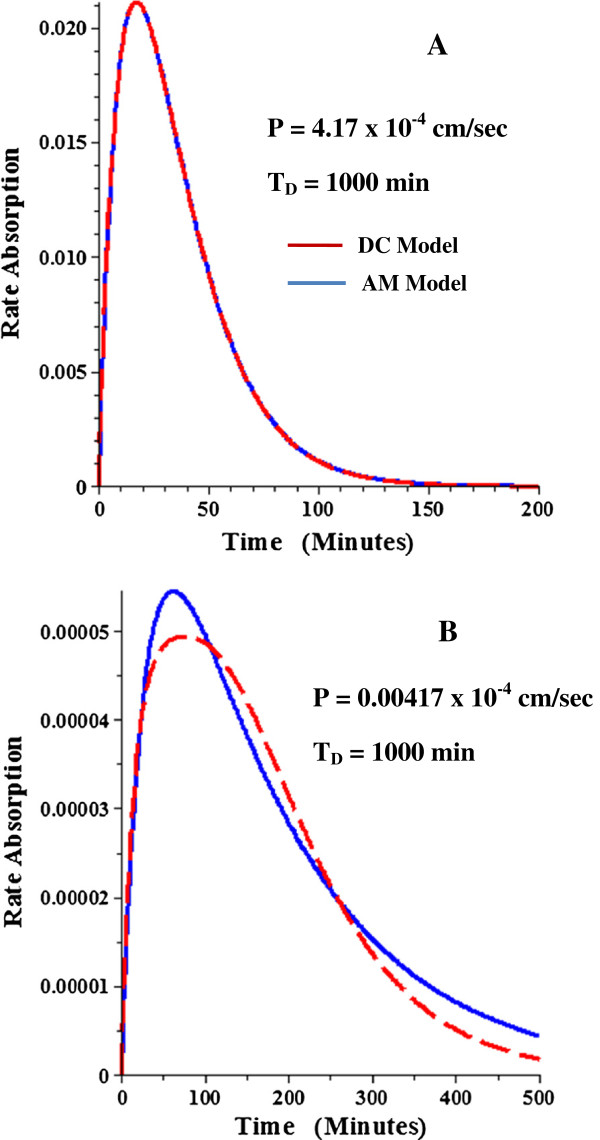
**Comparison of intestinal absorption rate for DC and AM models with T**_**D**_ **= 1000 minutes.** See Figure 8 for details.

The procedure that will be used to measure the experimental permeability is to fit the AM absorption rate Equation (15) to the systemic absorption rate determined by deconvolution. The accuracy of this procedure will be theoretically tested here by fitting Equation 15 to the general DC model absorption rate and comparing the AM permeability to the permeability that is used to generate the DC absorption data. Figure 8A and B compare the absorption rate as a function of time for the DC model (red) versus the best fit AM model (blue) for the case where the DC T_D_ = 200 minutes and T_F_ = 240 minutes (and T_G_ = 15 min, r = 1 cm, L = 700 cm). Figure 8A shows that the AM model provides a nearly perfect fit to the DC absorption rate for the case of a relatively high permeability solute (T_P_ = 200 min, corresponding to P = 4.167 × 10^-4^ cm/sec). This is expected because only 1% of the solute passes from the small to large intestine for this high permeability and, as shown above, the AM and DC models are theoretically identical if all the solute is absorbed. More surprisingly, the DC and AM models are nearly identical (Figure 8B) even for solutes with a very low permeability (P = 0.004167 × 10^-4^ cm/sec) where only 1% of the solute is absorbed. If the dispersion time constant (T_D_) is increased to 1000 minutes, the AM model provides a poorer approximation to the DC absorption rate for solutes with a low permeability (Figure 9B).

The quantitative comparison between the DC parameters used to generate the absorption rate and the AM parameters (T_G_, T_P_, and F_A_) that provide an optimal fit to this DC absorption rate are listed in Table 1 for a large range of values of the DC parameters. For T_D_ = 200 min, the AM permeability is within 20% of the DC permeability for a thousand fold permeability range (total absorption varying from 1% to 99%). For T_D_ =1000 min, the AM permeability can reach values 60% greater than the DC permeability for very low permeability solutes. Since the normal human T_D_ is about 200 minutes (Results, previous section), these results show that the AM model provides a good approximation to the exact DC model for a wide range of permeabilities.

**Table 1:** **Comparison of** “**averaged**” (**AM**) **and dispersion convection** (**DC**) **absorption rates**

**DC Model**				**AM Model**		
**P**_**DC**_** (10**^**-4**^** cm/sec)**	**T**_**D**_** (min)**	**T**_**G **_**(min)**	**Fr. Absorb**	**P**_**AM **_**(10**^**-4**^** cm/sec)**	**T**_**G**_** (min)**	**Fr. Absorb**
4.167	200	15	.987	4.77	17.2	.989
0.4167	200	15	0.600	0.493	18.56	0.604
0.04167	200	15	0.109	0.0514	19.76	0.111
0.004167	200	15	0.0119	0.00517	19.9	.012
4.167	1000	15	0.999	4.167	15	0.999
0.4167	1000	15	0.658	0.522	20.8	0.690
0.04167	1000	15	0.111	0.0645	29	0.121
0.004167	1000	15	0.0119	0.0067	30	0.0129
4.167	200	60	0.997	4.155	60.0	0.997
0.04167	200	60	0.109	0.062	86	0.107
4.167	1000	60	0.999	4.167	60.0	0.999
0.04167	1000	60	0.112	0.0879	114.5	0.121

The theoretical time dependent absorption rate was generated for the DC parameters listed in the table (P_DC_ = permeability, T_D_ and T_G_ = the dispersion and gastric emptying time constants). For all results, T_F_ = 240 min, r = 1 cm, L = 700 cm. The DC “Fr. Absorb” is the resultant cumulative fraction absorbed. The AM parameters (permeability (P_AM_), T_G_ and Fr. Absorb) were then adjusted to obtain the optimum fit to the DC absorption rate, similar to the procedure used to determine the experimental human small intestinal permeability by deconvolution.

### AM model estimates of the human intestinal permeability of 90 solutes

The Excel Table in the Additional file 1: Table 2 lists the values of the intestinal permeability for 90 solutes determined using the AM model and deconvolution. As discussed in the Methods there are two time constants in the AM model. For most of the solutes in this table, an oral solution was administered to fasting subjects so that the value of T in the range of 10 to 20 minutes can be assumed to be T_G_. For the few solutes in the table in which a tablet or capsule was administered, the solute had such a low permeability that it was clear that the longer T must correspond to T_P_.

In order to determine the permeability it is essential to relate the rate of solute absorption into the systemic circulation determined by deconvolution to the rate of intestinal absorption and this requires estimates of the liver and intestinal first pass extraction (Equation (15)). The liver extraction was determined from the estimated liver blood flow and the liver clearance (Equation (16)). The liver clearance is equal to the total systemic clearance (determined from the IV input blood data) corrected for the fractional renal clearance. These values are listed in Additional file 1: Table 2 for each solute. The value for the liver flow is just an estimate and for some drugs, e.g. β-blockers, the value is reduced. The intestinal extraction is more uncertain. Although certain drug classes are known to have significant intestinal metabolism, there is no quantitative data available in humans [23]. In Additional file 1: Table 2 the column labeled “Est Fraction Absorbed” represents the final estimate taking account of the best guess for intestinal extraction.

Three representative examples of AM model deconvolution calculations will be described in detail. Acetaminophen is the classic example of a high permeability drug. Its intestinal absorption rate is usually assumed to be so fast that its absorption rate is a measure of the rate limiting gastric emptying [24-26]. The deconvolution results shown in Figure 1 are based on the data of Ameer et. al. [27] for a 650 mg IV and oral (elixir) dose (data for one “representative” subject). Figure 1A shows the 2 exponential response function fit to the IV input data. Figure 1B compares the AM model prediction of the blood concentration with the experimental data for the oral dose, and Figure 1C shows the cumulative predicted absorption rate. The AM parameters are M = 545 mg, and the two time constants are 2 and 14 minutes. As discussed above, it is assumed that the time constant closest to 15 minutes is T_G_, and therefore T_P_ = 2 minutes. Correcting M for the liver extraction (Equation (16)) yields a fraction absorbed of 1.07; i.e. 100% absorption which is expected given the fact that the amount absorbed reaches its maximum by 50 minutes (Figure 1C), well before one would expect a significant amount to pass into the large intestine. From Equation (13), assuming an r of 1 cm, the acetaminophen permeability P_M_ is 41.7 × 10^-4^ cm/sec. There are two other published sets of acetaminophen data that can be used to estimate the permeability by deconvolution. The data of Divoll et. al. [28] (650 mg oral elixir data for representative “elderly” subject) has a P_M_ of 54 × 10^-4^ and that of Eandi et. al. [29] (averaged data (n = 9) for 1 gm oral “drops”) has a P_M_ of 12.6 × 10^-4^ cm/sec.

Risedronate is a pyridinyl bisphosphonate with a very low intestinal permeability (bioavailability < 1%). Despite this low permeability, the plasma pharmacokinetics described by Mitchell et. al. [30] after an oral (30 mg solution) and IV infusion (0.3 mg) can be used to determine the time course of intestinal absorption by deconvolution (Figure 2). The AM model provides an excellent fit to the oral plasma data (Figure 2B) with M = 220 mg (= 0.73% of 30 mg oral dose), T_G_ = 14 and T_P_ = 79.4 minutes. Using the fraction absorbed of 0.0073 in Equation (13), P_M_ = 0.008 × 10^-4^ cm/sec. (Since risedronate is not metabolized [30], there is no significant first pass metabolism.) The absorption is complete by 300 minutes (Figure 2C) presumably because this is the time required for complete emptying into the large intestine. This result also suggests that there is no significant absorption from the large intestine.

First pass intestinal extraction cannot be quantitatively measured in humans. In Additional file 1: Table 2 the assumed intestinal metabolism is indicated by the difference between the estimated total absorption (the column labeled “Est Fract Abs Small Intestine”) and the systemic absorption corrected for the liver extraction (column labeled “Fract Abs Corrected for Liver Clearance”). For example, cimetidine has a highly variable bioavailability of about 65% that has been attributed to either low intestinal permeability or intestinal metabolism [31]. The AM model provides a good fit (Figure 3A) to the blood concentration following a 300 mg oral solution dose [32]. The AM parameters are M = 175 mg, T_G_ = 10 and T_P_ = 25 minutes. Correcting for liver extraction raises the amount absorbed to 203 mg (68% of the oral dose). From the AM model time course of the amount absorbed (Figure 3B) it can be seen that the absorption is complete by about 100 minutes. This is short compared to the presumed small intestinal transit time of about 300 minutes, suggesting that permeability is not limiting and that intestinal metabolism is responsible for the incomplete absorption. This approach of assuming that permeability is not rate limiting if the absorption is completed in, e.g., 150 minutes can be used as a general criteria for determining if intestinal metabolism is important. (Note: this criteria is not applicable to acidic drugs, see Discussion). The extreme example of this for the drugs in Additional file 1: Table 2 is domperidone for which as much as 63% may be cleared by intestinal metabolism [33]. Cimetidine and domperidone are exceptions and for most of the drugs in Additional file 1: Table 2 intestinal metabolism is not significant.

## Discussion

As shown above (Results, Comparison of DC and AM models), the 3 parameter averaged model (AM) provides a good estimate of the small intestinal permeability if the following 2 conditions are met: 1) gastric emptying can be described by a single exponential process; and 2) the assumptions underlying the diffusion-convection (DC) model are valid. In addition, to convert the AM value of T_P_ to an absolute permeability requires an assumption about the small intestinal radius (r, Equation (13), assumed = 1 cm). The analysis listed in the Additional file 1: “Table 2” is limited primarily to drugs that were administered as oral solutions to fasting subjects, conditions for which the exponential emptying should be a good approximation [14]. The basic assumption of the DC model is that the small intestine can be described by a uniform volume cylinder with convective flow into each segment exactly balanced by flow out, combined with a mixing dispersion term, with all properties uniform for its entire length. This is, at best, an approximate description of the small intestine. Little is known about the details of small intestinal volume, mixing and dispersion in a fasting human subject that has swallowed the small volume of water (about 200 ml) that is usually administered in these oral solution dose studies.

Probably the most severe limitation of the DC model is the assumption that the parameters do not vary over the length of the intestine. The luminal pH definitely varies with position and, since the permeability of weak acids and bases depends critically on pH, this implies that their permeability will also vary with position. There have been a number of measurements of the pH position dependence of the human intestine. In a review of the older literature, Gray and Dressman [34] reported pH values of 4.9 in proximal duodenum, 5.3 in terminal duodenum, 4.4-6.5 in proximal jejunum, 6.6 in mid and terminal jejunum and varying from 6.5 in proximal ileum to 7.4 in terminal ileum. Using in situ pH microelectrodes Ovesen et. al. [35] simultaneously measured a fasting pH of 2.05 in stomach, 3.03 in duodenal bulb, 4.9 in mid duodenum and 4.92 in proximal jejunum. Using radiotelemetry capsules swallowed “with a small quantity of water”, Evans et. al. [36] reported pH values of 6.63 in jejunum, 7.41 in mid small bowel, 7.49 in ileum and from 6.37 to 7.04 in colon. Using the “smart pill”, Lalezari [37] recently reported pH values varying from 5.6, 6.2, 6.68, 6.9 for proximal to terminal small intestinal quartiles. Thus, the small intestinal pH can be assumed to start at about 4.4 in an initial short segment of the duodenum, increasing to 5.4 in the first part of the jejunum, to 6.4 in mid intestine and to 7.4 in the terminal ileum.

The results in Additional file 1: Table 2 will be discussed in terms of the classical pH partition assumption that the permeability is proportional to the concentration of the neutral moiety, using the octanol/water partition (log D) as representative of the epithelial membrane partition and the pKa to estimate the neutral concentration [38]. Since the small intestinal pH varies from about 4.4 to 7.4, this pH partition hypothesis implies that the intestinal permeability can vary by as much as 1000 fold over its entire length. Although more complicated approaches that combine log D with estimates of polar surface area and hydrogen bond donors can improve permeability estimates [39], log D captures the main features and will be focused on here. It is hoped that the data set in Additional file 1: Table 2 will be used in future evaluations of these advanced models.

**Figure 10:**
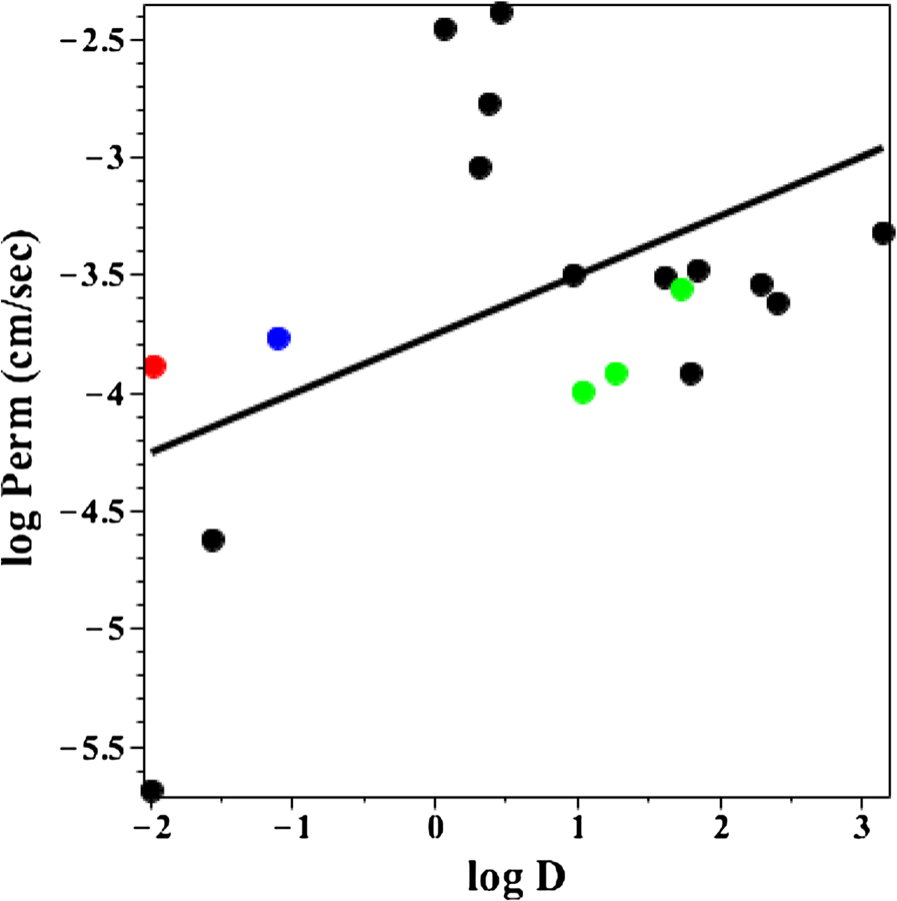
**Permeability versus octanol partition for neutral solutes.** Plot of the log of the permeability (cm/sec) versus the log of the octanol/water partition (log D) for the neutral solutes in Additional file 1: Table 2. The colored points indicate solutes which may have protein mediated transport: Red = xylose (glucose transport system); Green = digoxin, β-methyl digoxin and colchicine (P-glycloprotein mediated efflux); Blue = lamivudine (organic cation transporter). The solid line is the linear regression fit to the black points.

Since the permeability of the 18 uncharged solutes in Additional file 1: Table 2 should not be affected by this pH heterogeneity, one would predict that the AM permeability for these solutes should be a good approximation to the true permeability. Figure 10 shows a plot of the log D versus the log of the permeability (cm/sec). The 5 colored points indicate solutes for which there is strong evidence of protein mediated transport. The 3 green points have P-glycloprotein mediated efflux (digoxin and β-methyl digoxon [40] and colchicine [41]) which should reduce their permeability. The blue point is lamivudine which is a substrate for the organic cation transporters [42] and the red point is xylose which has a carrier mediated transport (probably the glucose transport system) [43-45], both of which will increase the “permeability”. The black line is the least squares regression fit to the black (non-protein mediated) points. There is only a weak correlation between permeability and log D. Presumably other factors besides simple octanol partition are important. The 4 points with the highest permeability (caffeine, acetaminophen, antipyrine and cotinine) are all low molecular weight (< 200) solutes. The 2 points with log D < -1.5 (acyclovir and ganciclovir) may have significant paracellular transport.

**Figure 11:**
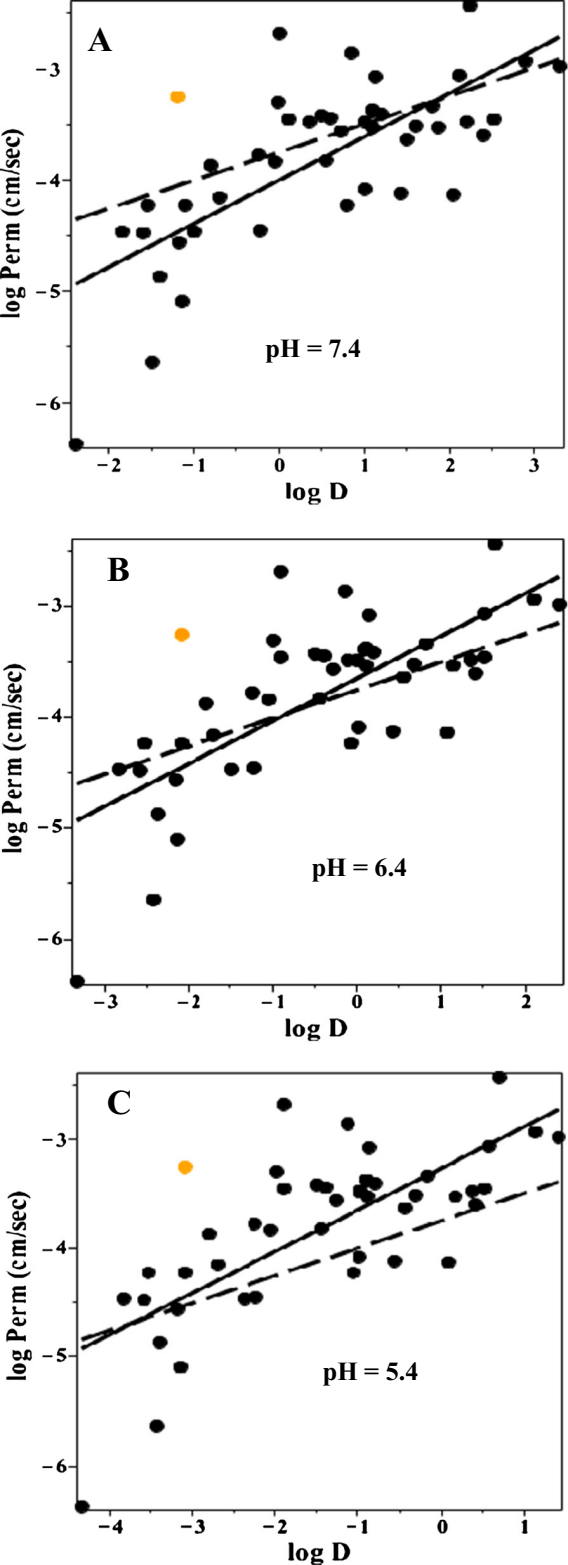
**Permeability versus octanol partition for basic solutes.** Plot of the log of the permeability (cm/sec) versus the log of the octanol/water partition (log D) for the basic solutes in Additional file 1: Table 2. The log D was determined at pH = 7.4 (Figure 11**A**), 6.4 (Figure 11**B**) and 5.4 (Figure 11**C**). The solid line is the linear regression fit to the black points and the dashed line is the linear regression fit to the neutral solutes (Figure 10). The orange point is midodrine which is a substrate for the peptide transport system.

One would predict that the small intestinal pH heterogeneity should significantly influence the apparent AM “permeability” of the basic solutes listed in Additional file 1: Table 2. Since the basic solutes have a higher uncharged concentration at the higher pH, they will tend to be absorbed in the terminal small intestine – delaying their absorption and decreasing the calculated permeability. This delay should have a complicated dependence on log D. Solutes with a relatively high log D at pH 6.5 will be absorbed in the first part of the intestine and have a correspondingly higher apparent permeability then solutes with a low log D whose absorption will be delayed until they reach the pH of 7.4 in the ileum. Figure 11 shows a plot of log D versus log permeability for the basic solutes in Additional file 1: Table 2 for a log D determined at pH 7.4 (Figure 11A), pH 6.4 (Figure 11B) and pH 5.4 (Figure 11C) using Equation (20) to convert the log D to the different pHs. The orange point is midodrine which is a known substrate for the peptide transport system [46]. The solid line is the linear regression fit to the black points and the dashed line is the regression for the neutral solutes (Figure 10). The pH 6.4 plot provides the best fit to the neutral solute permeability data (which should not be pH dependent), suggesting that pH 6.4 is the best average approximation for basic solutes. This is consistent with the current recommendation to use a pH of 6.8 for studies of “simulated” intestinal fluid [34]. As predicted, the AM permeability of the basic solutes with low log D at pH 6.4 or 7.4 is less than that of the neutral solutes (dashed line) because their absorption should be delayed until they reach the ileum. This comparison between the neutral and basic solutes is only suggestive because of the small number of neutral solutes in Additional file 1: Table 2 and their poor correlation with log D (Figure 10).

The opposite effect should occur for the acidic solutes which should be absorbed in the proximal (acidic) section of the intestine. The classic example is aspirin, which has a pKa of 3.49 and a log D of about -1.8 (average from LOGKOW) at pH 7.4. From the plots in Figures 10 or 11, one would predict that a solute with this log D should have a low permeability of about 0.4 × 10^-4^ cm/sec, about 25 times smaller than the experimental AM aspirin permeabilities (Additional file 1: Table 2) of 6.69 × 10-4 cm/sec (Rowland et al. [47] for one subject) or 20.8 × 10^-4^ cm/sec (Bochner et al. [48], average of 6 subjects). The explanation of this high permeability has been controversial. Hogben et al. [49] used this rapid absorption of aspirin to infer that there must be a pH of about 5.3 at the luminal surface of the epithelial cell maintained by some unknown mechanism combined with a large unstirred luminal fluid layer. However, the recognition that the unstirred layer in humans is only about 35 μm [50] makes this idea untenable and direct measurements in guinea pig jejunum do not find evidence for this acidic mircroclimate [51]. An alternative explanation is that the salicylates are transported by a monocarboxylic acid carrier system [52,53]. However, Takagi et al. [54] suggested this result is an artifact and that pure phospholipid liposomes show the same apparent “carrier” behavior. The most likely explanation is simply that aspirin is absorbed in the duodenum and proximal jejunum where the pH varies from 4.4 to 5.4. At a pH of 5.4, the log D of aspirin is about 0.19 (Equation (20)) and small neutral solutes with this log D (e.g. caffeine, see Figure 10) have high AM permeabilities, equal to or greater than are observed for aspirin. The aspirin permeability at pH 5.4 is presumably high enough that it can be nearly completely absorbed in this short proximal region.

A dramatic illustration of the effect of this pH heterogeneity on the absorption of weak acids is provided by acetylcysteine which has a pKa of 3.25 and a very low log D of -2.5 at pH 7.4 with a corresponding log D of -1.5 at pH 6.4 and -0.6 at pH 5.4. The AM fit to the blood concentration following the oral dose and the time course of the intestinal absorption is shown in Figure 4. Even though the permeability time constant T_P_ is very fast (6.95 minutes), only about 12% of the 3676 μm oral dose is absorbed and the absorption stops after about 50 minutes (Figure 4B). This suggests that the absorption occurred only in the low pH proximal small intestine and this region was cleared by about 50 minutes after 12% was absorbed and that there was no significant absorption in the rest of the intestine.

**Figure 12:**
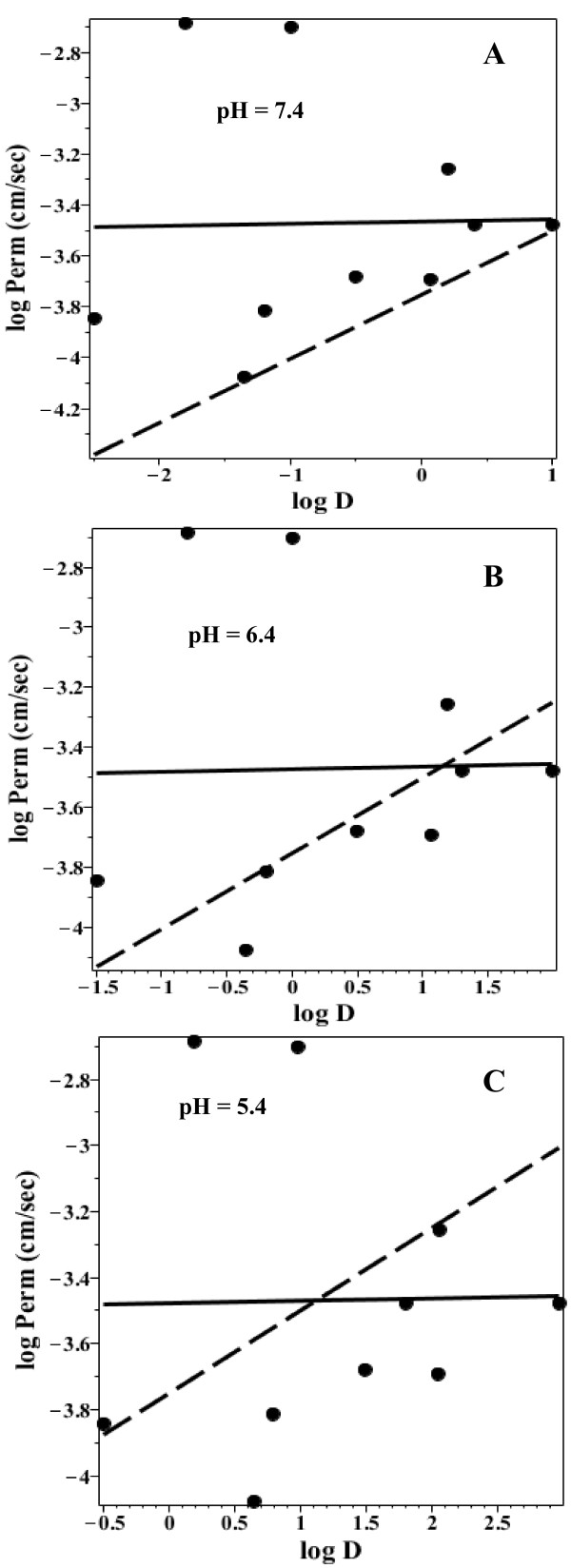
**Permeability versus octanol partition for acidic solutes.** Plot of the log of the permeability (cm/sec) versus the log of the octanol/water partition (log D) for the acidic solutes in Additional file 1: Table 2. The log D was determined at pH = 7.4 (Figure 12**A**), 6.4 (Figure 12**B**) and 5.4 (Figure 12**C**). The solid line is the linear regression fit to the black points and the dashed line is the linear regression fit to the neutral solutes (Figure 10).

Figure 12 shows a plot of the log permeability versus the log D at pH 7.4 (Figure 12A), 6.4 (Figure 12B) and 5.4 (Figure 12C) for the 10 acidic solutes in Additional file 1: Table 2. The solid line is the linear regression fit to the black points and the dashed line is the regression for the neutral solutes (Figure 10). There is no significant correlation between log D and the permeability. This is probably because most of these solutes are relatively rapidly and nearly completely absorbed in the proximal intestine and the log D varies over a smaller range than the basic solutes (Figure 11).

**Figure 13:**
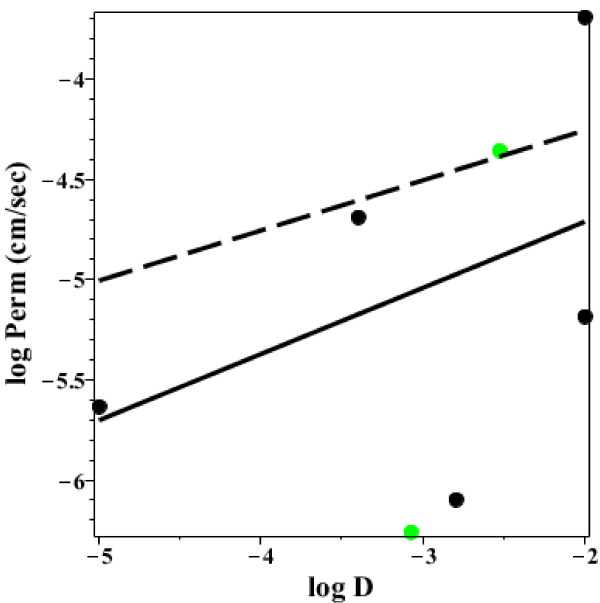
**Permeability versus octanol partition for charged solutes.** Plot of the log of the permeability (cm/sec) versus the log of the octanol/water partition (log D) for the charged solutes in Additional file 1: Table 2. The solid line is the linear regression fit to the black points and the dashed line is the linear regression fit to the neutral solutes (Figure 10). The green points are cefixime and aztreonam which are substrate for the P-glycloprotein mediated efflux system.

Figure 13 shows a plot of the log permeability versus the log D for the 7 solutes in Additional file 1: Table 2 that are charged over the entire pH range 5.4 to 7.4. The 2 green points indicate solutes that may be substrates for the peptide transport system. All the solutes have low permeabilities that are less than are predicted by the neutral solute plot (dashed line). These solutes are probably absorbed primarily by paracellular transport.

**Figure 14:**
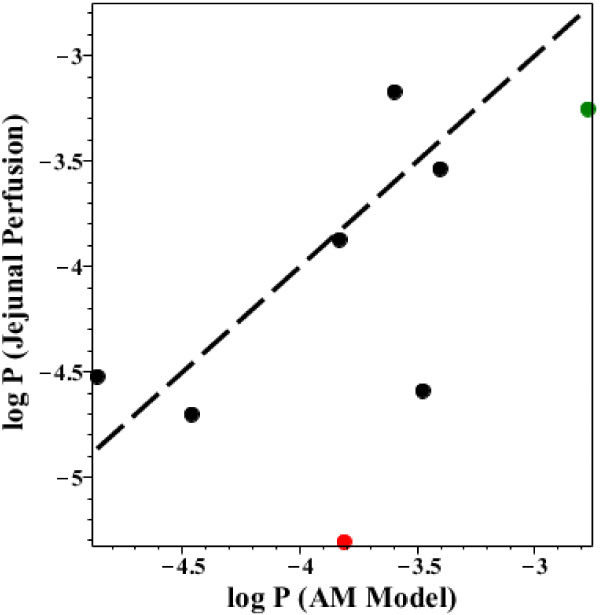
**Comparison of the AM versus the human jejunal perfusion permeability.** Plot of the log of the jejunal permeability (cm/sec) versus the log of the AM permeabililty (Additional file 1: Table 2). The dashed line is the line of identity. The red point is the weak acid furosemide and the green point is the uncharged antipyrine.

The best currently available measurements of human small intestinal permeability are the single-pass jejunal perfusion results of Lennernas and colleagues. Currently, they have published the jejunal permeability for 28 drugs [18]. Figure 14 shows a log-log plot of the AM versus the perfused jejnunal permeability for the 8 drugs that were studied by both methods. The dashed line is the line of identity. The black and red points are weak bases and acids, respectively, and the green point is the uncharged solute antipyrine. It can be seen that for most solutes the AM permeability is in good absolute agreement with the direct perfusion permeability - a surprising result considering the marked differences in experimental approaches and assumptions for the two methods. The major exception is the weak acid furosemide (red point) whose AM permeability (1.54 × 10^-4^ cm/sec) is 30 times greater than the perfusion permeability, presumably because furosemide is absorbed primarily in the proximal jejunum that has a pH significantly more acid than the pH of 6.5 used in the perfusion studies.

One can use the AM permeability of the highest permeability solutes to estimate a lower bound for the unstirred aqueous layer. For the passively absorbed high lipid solubility drugs, the permeability (P) should be approximately equal to that of the total fluid layer separating the intestinal capillaries from the well stirred lumen:

(21) $$P=D_{\mathrm{US}}/L_{\mathrm{US}}$$

where L_US_ is the thickness and D_US_ is the average diffusion coefficient for this fluid layer. The small uncharged solutes (e.g. caffeine, acetaminophen and antipyrine) have the highest AM permeabilities of about 20 × 10^-4^ cm/sec (Additional file 1: Table 2). Assuming a D of 9.1 × 10^-6^ cm^2^/sec for, e.g., antipyrine in water at 37°C [55], L = 45 μm. Since the epithelial cell thickness is about 25 μm [56], this corresponds to an unstirred luminal layer of only about 20 μm, similar to the value of 35 μm found by Levitt et al. [50] for human jejunum. This AM antipyrine permeability value is about 3 times larger than the value found by Fagerholm and Lennernas [55] at the highest rates of jejunal perfusion. The perfusion at a pressure of about 20 mm Hg [57] produces an unphysiological distended jejunum (radius of 1.61 cm) [58] and one might expect greater unstirred layers than during the nearly fasting conditions used for the AM studies.

**Figure 15:**
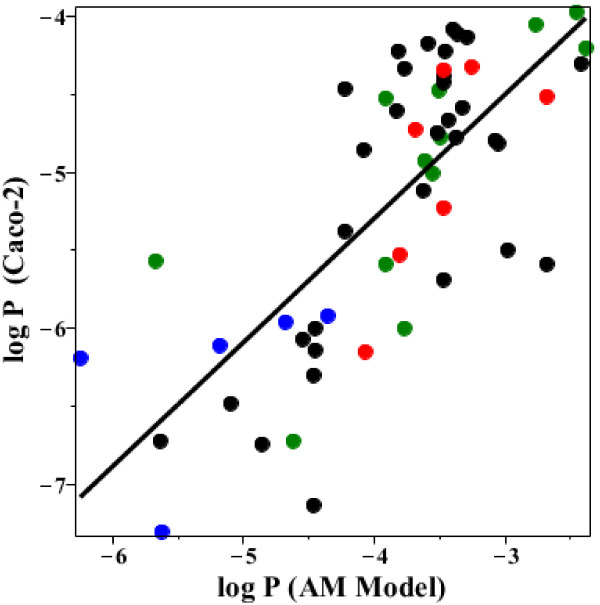
**Comparison of the AM versus the Caco**-**2 permeability.** Plot of the log Caco-2 permeability (cm/sec) versus the log of the AM permeability (Additional file 1: Table 2). The points are colored on the basis of their charge state: Black = basic, Red = acidic, Green = uncharged, Blue = charged. The solid line is the linear regression fit to all the points.

The standard procedure for screening for the intestinal permeability of drugs is the Caco-2 cell culture system. Comparison of the AM permeability with the Caco-2 permeability is inexact because of the variety of techniques that have been used for reported Caco-2 values, with results differing by as much as 10 fold between different labs [1]. Larregieu and Benet [59] recently reviewed some of the problems in using Caco-2 as a surrogate for human permeability measurements. Thomas et al. [60] recently published a compilation of results for 120 drugs determined in their lab by the same method and these values were compared with the AM values for the drugs studied by both methods (Additional file 1: Table 2). In addition, Additional file 1: Table 2 was filled in with Caco-2 results from other labs. When more than one value was available, usually the larger permeability was used. Figure 15 shows a log-log plot of the AM versus Caco-2 permeability. The solid line is the linear regression fit. At the high permeability end of the regression, the AM permeability is about 40 times greater than the Caco-2 permeability. This is consistent with a Caco-2 unstirred layer that varies, depending on the stirring rate, from 564 to 2500 μm [61], which is 12 to 55 times greater than the AM value. At the low permeability end where the unstirred layer is not limiting, the AM permeability is 6.8 times greater than the Caco-2 permeability.

**Figure 16:**
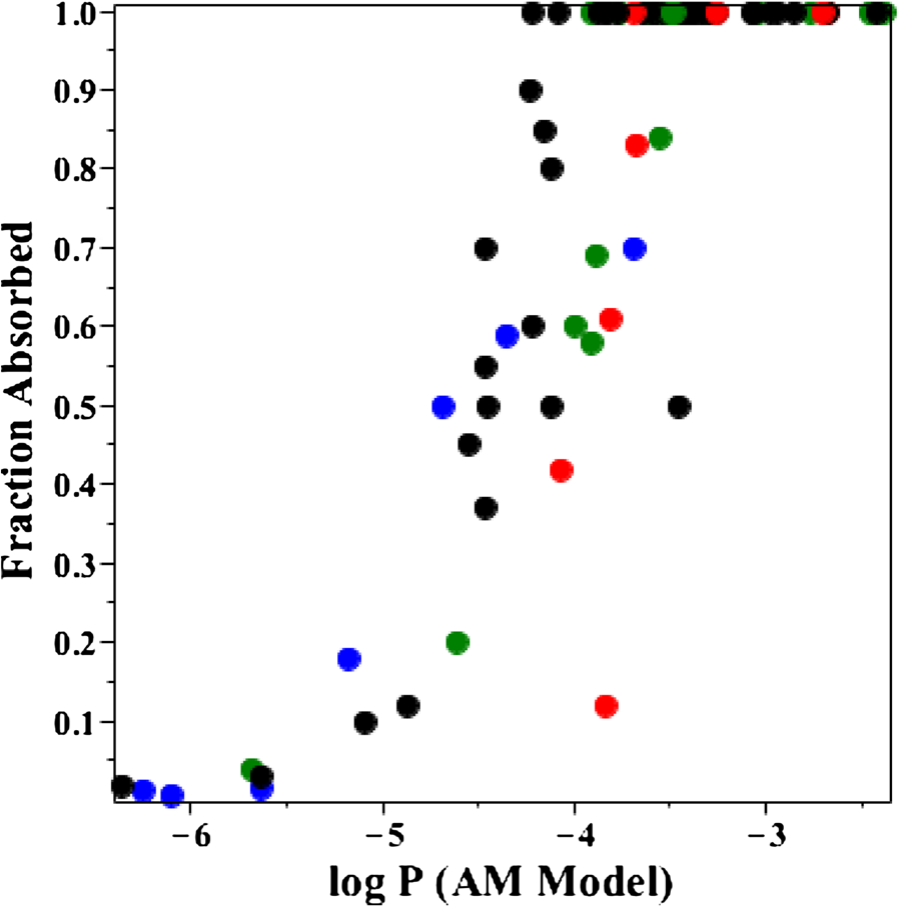
**AM fraction absorbed versus AM permeability.** Plot of the AM fraction absorbed versus the log of the AM permeability (cm/sec). The points are colored on the basis of their charge state: Black = basic, Red = acidic, Green = uncharged, Blue = charged.

As discussed in the Background section, the standard approach for evaluating QSAR predictions of intestinal drug permeability is to use the human fraction absorbed as a surrogate for the permeability. Although the limitations of this approach are well recognized [1], it is the only available correlate of absorption for most drugs. The plot of the fraction absorbed versus the log of the AM permeability for the drugs in Additional file 1: Table 2 (Figure 16) dramatically illustrates this limitation. For values of the AM P greater than about 10^-4^ cm/sec, the drugs are 100% absorbed and, for P less than about 10^-5^, absorption drops to 10% or less. Thus, although the permeability varies over a 10,000 fold range, the fraction absorbed varies from 0.1 to 1 over just a 10 fold permeability range. Although the fraction absorbed is the clinically most important prediction, it would clearly be useful to be able predict the permeability over a wider range. The data in Additional file 1: Table 2 should provide a useful benchmark for QSAR analysis.

## Conclusions

The “averaged model” (AM) model accurately describes intestinal absorption if the assumptions of the diffusion convection (DC) model are satisfied. This new simple 3 parameter function (Equation (15)) can be used to determine by deconvolution the human intestinal permeability during the normal human drug absorption process. The AM permeability is similar to the values measured using direct jejunal perfusion. Its main limitation results from the heterogeneity in the small intestinal permeability of weak acids and bases produced by the variation in intestinal pH. Weak acids will tend to be absorbed in the proximal intestine and weak bases in the terminal intestine and this will be represented in the “permeability” determined by this method. The permeability data for the 90 drugs described in the Additional file 1: “Table 2” provides a large data base that should be useful in drug development and QSAR analysis.

## Abbreviations

AM: Averaged model; DC: Diffusion convection model; D: Dispersion coefficient; DUS: average unstirred layer diffusion coefficient; LUS: Unstirred layer thickness; F: Intestinal convective flow; P: Permeability for DC model; PM: AM permeability for case where F_A_ of dose is absorbed; R: Intestinal radius; L: Intestinal length; S: Surface area = 2πrL; V: Volume = πr^2^L; N: Number of finite segments in numerical solution; ∆P: PS/N; ∆V: V/N; De: πr^2^DN/L; Dose: Total oral dose; FA: Fraction of dose absorbed; IG(t): Convective solute input from stomach; EDC: DC flux from small to large intestine; ADC(t): DC cumulative amount leaving small intestine; AM: AM cumulative amount absorbed for case where F_A_ of dose is absorbed; EH: Fractional liver extraction; EI: Fractional intestinal mucosal extraction; ClH: Liver clearance; QH: Liver blood flow; M: Amount absorbed = F_A_ Dose; MS: AM amount entering the systemic circulation; RDC: DC rate of intestinal absorption; R: AM rate of intestinal absorption for case where 100% absorbed in small intestine; RM: AM rate of absorption for case where F_A_ of dose is absorbed; RSM: AM rate of absorption corrected for intestinal and liver extraction; c(x,t): DC concentration at position x at time t; C(t): “averaged” AM concentration; Coral: Experimental blood concentration following an oral dose; TF: DC convective time constant; TD: DC dispersion time constant; Tp: Intestinal permeability time constant; TG: Gastric emptying time constant; pKa: Acid dissociation constant; Pow: Octanol/water partition; log D: log P_ow_ at pH = 7.4.

## Competing interests

The author declares that he has no competing interests.

## Authors’ contributions

DGL is the sole contributor to this work.

## Pre-publication history

The pre-publication history for this paper can be accessed here:

http://www.biomedcentral.com/2050-6511/14/34/prepub

## Supplementary Material

Additional file 1: Table 2 Summary of averaged model (AM) deconvolution analysis of human intestinal absorption. Tabulated summary of all the permeability data used in the paper “Quantitation of small intestinal permeability during normal human drug absorption”, D. G. Levitt. Click here for file
